# Same Organ, Two Cancers: Complete Analysis of Renal Cell Carcinomas and Upper Tract Urothelial Carcinomas

**DOI:** 10.3390/medicina60071126

**Published:** 2024-07-12

**Authors:** Sorin Vamesu, Oana Andreea Ursica, Serban Eduard Milea, Mariana Deacu, Mariana Aschie, Anca Florentina Mitroi, Felix Voinea, Mihaela Butcaru Pundiche, Cristian Ionut Orasanu, Raluca Ioana Voda

**Affiliations:** 1 Clinical Service of Pathology, Departments of Pathology, “Sf. Apostol Andrei” Emergency County Hospital, 900591 Constanta, Romania; 2 Faculty of Medicine, “Ovidius” University of Constanta, 900470 Constanta, Romania; 3 Department of Anatomy, Academy of Medical Sciences of Romania, 030171 Bucharest, Romania; 4 Department of Medical Sciences, The Romanian Academy of Scientists, 030167 Bucharest, Romania; 5 Center for Research and Development of the Morphological and Genetic Studies of Malignant Pathology (CEDMOG), “Ovidius” University of Constanta, 900591 Constanta, Romania; 6 Clinical Service of Pathology, Departments of Genetics, “Sf. Apostol Andrei” Emergency County Hospital, 900591 Constanta, Romania; 7 Urology Clinical Department, “Sf. Apostol Andrei” Emergency County Hospital, 900591 Constanta, Romania; 8 Clinical Department of General Surgery, “Sf. Apostol Andrei” Emergency County Hospital, 900591 Constanta, Romania

**Keywords:** renal cell carcinoma, upper tract urothelial carcinoma, perineural invasion, risk factor, hematuria

## Abstract

*Background and Objectives*: Renal cell carcinomas and upper tract urothelial carcinomas are types of malignancies that originate in the kidneys. Each of these examples shows an increasing trend in the frequency and the mortality rate. This study aims to comprehensively define carcinomas by analyzing clinical, paraclinical, and histological aspects to predict aggressiveness and mortality. *Materials and Methods*: We conducted a retrospective investigation on a group of patients suspected of kidney cancers. *Results*: We identified 188 cases. We observed a higher mortality rate and older age in individuals with urothelial carcinomas. Anemia, acute kidney injury, hematuria, and perineural invasion were the main risk factors that predicted their mortality. Tumor size in renal cell carcinomas correlates with the presence of necrosis and sarcomatoid areas. Factors that indicate a higher rate of death are older age, exceeding the renal capsule, a lesion that includes the entire kidney, lymphovascular invasion, acute kidney injury, and anemia. *Conclusions*: Even if they originate at the renal level, and the clinical–paraclinical picture is similar, the histopathological characteristics make the difference. In addition, to these are added the previously mentioned common parameters that can represent important prognostic factors. In conclusion, the characteristics commonly identified in one type of cancer may act as risk factors for the other tumor. The detected data include threshold values and risk factors, making a significant contribution to the existing literature.

## 1. Introduction

Cancers originating from the kidneys are on the rise, with a not negligible mortality rate [1]. Over 90% of kidney cancers are renal cortical tumors. Among these, the most common histopathological type encountered is clear cell renal cell carcinoma (approximately 70% of cases). In the rest of the cases, the histomorphology highlights papillary, chromophobe, and collecting duct tumors [2,3]. Malignant tumors originating in the calyceal system and the renal pelvis are also called upper tract urothelial carcinoma (UTUC). These are represented by invasive urothelial carcinomas and low- and high-grade papillary urothelial carcinomas [4,5].

Renal cell carcinomas (RCCs) represent approximately 2.2% of all cancers, representing the seventh most prevalent type of cancer in developed countries. In recent decades, its incidence has increased from 7.1/100,000 to 14.9/100,000. International statistical reporting programs indicate mortality with an undulating distribution with an average rate of 3.6/100,000, representing approximately 1.8% of cancer deaths globally [6]. UTUCs represent less than 10% of renal cancers and approximately 5% of urothelial cancers. Their real incidence is difficult to determine, but they are often related to renal carcinomas. Studies show an incidence that varies between 1.15/100,000 and 2.06/100,000 patients per year [7]. Data on mortality are scarce, but population studies report a 5-year mortality rate of approximately 25% [8]

In the case of RCC, the diagnosis based on symptoms and physical examination is limited. Only 30% of patients receive a diagnosis based on the symptomatic triad of flank pain, hematuria, or a palpable abdominal mass. As a rule, the presence of symptoms is associated with aggressiveness, and aspects such as bone pain, persistent cough, or alteration in performance status indicate an advanced stage [9,10]. Most frequently, these tumors are diagnosed by imaging. The most common methods are computed tomography or magnetic resonance imaging. Their role is to describe the tumor mass, extension, venous involvement, as well as possible metastases [9]. Similarly, the clinical manifestations of UTUC are, in most cases, nonspecific. The most common aspects are represented by hematuria (70–80% of patients), flank pain (20% of patients), and/or palpable lumbar mass (10% of cases). Signs that suggest an advanced stage are night sweats, weight loss, fever, cough, or anorexia [11]. The standard investigation is urography by computed tomography. This has increased specificity and describes a filling defect, an obstruction, or an incomplete filling of the upper tract. Only in the case of patients with contraindications is magnetic resonance urography recommended [11,12].

Until now, studies have identified some risk factors involved in the development of these cancers. In the case of RCCs, the risk is attributed to advanced age, obesity, smoking, high blood pressure, and increased consumption of analgesics [13,14]. For UTUC, four factors present an increased risk in their development: smoking, alcohol, aromatic amines, and consumption of products rich in aristolochic acid [11]. Regarding the prognostic factors that play a role in patient mortality, research has not reached clear, fully elucidated data. The most frequently found risk factors predicting aggressiveness and mortality in RCCs are represented by the ECOG scale, advanced stage, nodal invasion, histological grade, tumor size, and vascular invasion [15,16]. On the other hand, the most common parameters in UTUCs are tumor stage (>T2), high grade, lymph node invasion, and positive surgical margins [17,18].

Given these data, as well as the worrisome incidence and death rates, through this study, we want to conduct a more thorough analysis of these two pathologies, following demographic, clinical, paraclinical, morphometric, and histopathological parameters. Thus, this study aims to identify the factors that predict the aggressiveness of these types of cancer and are associated with death. Also, we aim to complete the information from the literature, which is possible through a brief review of it and by comparing the identified results.

## 2. Materials and Methods

**Figure 1:**
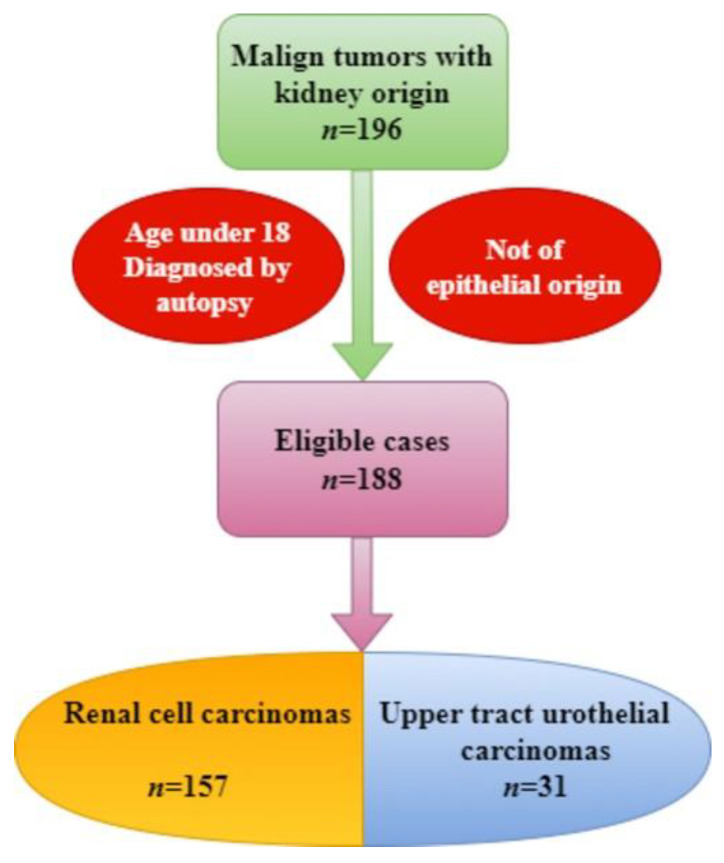
Flow chart with the batch studied.

We conducted a retrospective study for the period 1 January 2017–31 December 2023 of patients diagnosed with renal tumors at the Constanta County Emergency Hospital, Dobrogea. The data were extracted from the archives and electronic databases of the hospital. The inclusion criteria consisted of adult patients with a presumptive diagnosis of a malign tumor developed at the level of the kidney. The exclusion criteria consisted of patients under the age of 18, cases diagnosed by autopsy, and tumors that are not of epithelial origin (Figure 1).

The clinical and paraclinical aspects were extracted from the patients’ observation sheets. All patients underwent imaging examinations in the hospital. The excised specimens were described macroscopically according to the international protocols in force and processed up to the stage of the microscopic slide in the usual staining in hematoxylin–eosin in the Clinical Pathological Anatomy Service of Constanta. The histopathological diagnoses were reevaluated by two pathologists according to the latest WHO classification (*Urinary and Male Genital Tumours*, fifth edition, 2022).

The statistical analysis of the data was conducted using SPSS Statistics Version 26 (IBM Corporation, Armonk, NY, USA). We utilized indicators of central tendency and variability. For the analysis of univariate data, we utilized Fisher’s exact test for categorical data and both the Mann–Whitney U Test and the Kruskal–Wallis H test for continuous variables, as appropriate. To measure the association of the data, we used the Pearson correlation coefficient, and for the prediction of the response between variables, the Pearson regression. Receiver operating characteristic (ROC) and area under the curve (AUC) were used to establish the accuracy of the parameters. The sensitivity and specificity of the parameters are the optimal cut-off point as the value that maximizes the area under the ROC curve. To assess the hazard risk (HR), the binary logistic regression or linear regression test was used, as appropriate. The results reached statistical significance with a *p*-value of less than 0.05.

## 3. Results

### 3.1. Demographic Aspects

Following the inclusion and exclusion criteria, 188 cases of malignant epithelial tumors resulted, 31 starting from the pyelocalyceal system and 157 starting from the renal parenchyma.

**Figure 2:**
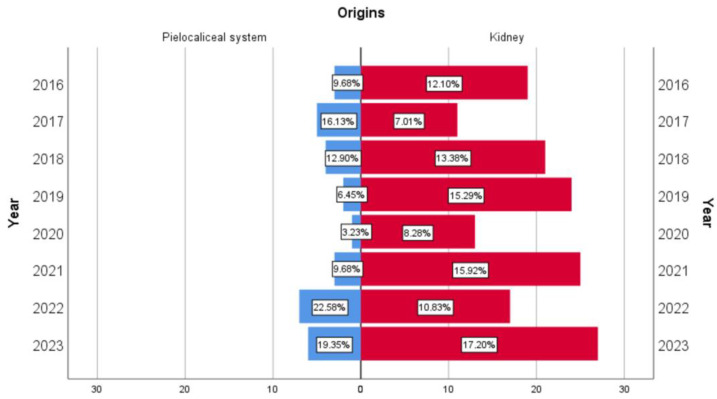
The undulatory distribution of the two types of cancers.

In both situations, the diagnostic trend was undulating. UTUC presented two incidence peaks in 2017 (16.13%) and 2022 (22.58%). In renal cell carcinomas, the trend showed several incidence peaks (Figure 2).

We observed a statistically significant difference between the ages of diagnosis and the point of origin (*p* < 0.001), as well as regarding death—UTUC having a higher mortality rate (*p* = 0.029) (Table 1). In the case of RCC, advanced age at diagnosis is associated with death (*p* = 0.001). Age ≥ 64.5 years has a sensitivity of 61.5% and specificity of 60.2% for death (AUC = 0.671, *p* = 0.001).

### 3.2. Clinical and Paraclinical Characteristics

Comparatively, in cases of renal cell carcinoma, the manifestations such as oligoanuria and hematuria are more attenuated (absent or macroscopic hematuria), the more frequent symptoms being manifested by flank pain. On the other hand, in UTUC, patients’ presentations are marked by anemic syndrome and acute renal injury (Table 2).

In RCC, we observed a difference between the presence of renal insufficiency and gender, this being more frequent in the case of the male sex (*p* = 0.039), and an increased average of serum creatinine is also observed for them (*p* < 0.001). The presence of hypertension is associated with other comorbidities such as diabetes mellitus (*p* = 0.038) and dyslipidemia (*p* < 0.001). Also, diabetes mellitus is associated with dyslipidemia (*p* < 0.001). Detection of acute kidney injury is associated with anemia (*p* = 0.001), without being associated with its degree (*p* = 0.702). As expected, anemia is associated with hematuria (*p* = 0.009). The presence of anemia correlates with the death rate (*p* = 0.011).

Acute renal injury is associated with systemic inflammatory syndrome (*p* = 0.004) and oligoanuria (*p* = 0.045). Also, this correlates with the death rate (*p* = 0.013). In the case of these patients, a preoperative level of serum creatinine ≥ 0.95 mg/dL has a sensitivity of 64.1% and a specificity of 66.9% for death (AUC = 0.647, *p* = 0.006). Increased creatinine levels are correlated with other paraclinical parameters such as systemic inflammatory syndrome (*p* = 0.001) and anemia (*p* = 0.002), but not with its degree (*p* = 0.200). In turn, the systemic inflammatory syndrome is correlated with the presence of oligoanuria (*p* = 0.005) and anemia (*p* = 0.045).

In cases of UTUCs, patients’ hypertension is associated with the presence of dysuria (*p* = 0.015). Obesity presents statistically significant correlations with the female gender, being even more common in them (*p* = 0.032). Acute renal injury is correlated with the presence of anemia (*p* = 0.003), inflammatory syndrome (*p* = 0.023), and macroscopic hematuria (*p* = 0.045). Also, elevated creatinine values are associated with the presence of anemia (*p* = 0.004) and inflammatory syndrome (*p* = 0.005). Macroscopic hematuria was associated with the death of patients, unlike the one discovered by the examinations performed after hospitalization (*p* = 0.026).

### 3.3. Morphometric Aspects

Comparatively, renal mass and length are increased in RCC compared to UTUC, but without statistical significance. In both categories, the left kidney is more frequently involved, the tumors being more often found in the upper third for tumors originating from the renal tubes epithelium and in the middle third (middle calyceal system and pelvis) in renal urothelial tumors (Table 3).

In RCC, we observed a difference between the weight of the kidneys and their size and gender. Thus, in the case of women, the weight and length are reduced more than in the case of men (*p* < 0.001 and *p* = 0.034, respectively).

In UTUC, a kidney with increased weight is correlated with the presence of anemia (*p* = 0.037) and with its moderate degree (*p* < 0.001). A renal mass of ≥242 g shows a sensitivity of 90.9% and a specificity of 66.7% for the presence of anemia in these patients (AUC = 0.741, *p* = 0.039).

### 3.4. Macroscopic Aspects

As expected, aspects such as tumor pseudoencapsulation and cystic spaces are the prerogative of renal cell carcinomas, while the infiltrative aspect is more frequently encountered in upper tract urothelial carcinomas (Table 4).

In RCC, the maximum diameter of the tumor was directly proportionally associated with kidney weight and length (*p* < 0.001 and *p* < 0.001, respectively). Moreover, we observed the fact that the tumor diameter is larger in the case of men (*p* = 0.012). The location of the tumor in the lower pole is associated with the invasive character in the pyelocalyceal system (*p* = 0.045) or the perirenal tissue (*p* = 0.011). As expected, the invasiveness of the pyelocalyceal system is associated with the lack of pseudoencapsulation (*p* = 0.035). Also, the absence of the capsule is associated with high T (>3a) staging (*p* = 0.004), as well as with the presence of acute renal injury (*p* = 0.023), with serum creatinine levels supporting this aspect (*p* = 0.036). Exceeding the renal capsule is associated with the presence of anemia (*p* = 0.031), oligoanuria (*p* = 0.017), and dysuria (*p* = 0.036). It is also associated with death (*p* = 0.049).

### 3.5. Microscopic Characteristics

**Figure 3:**
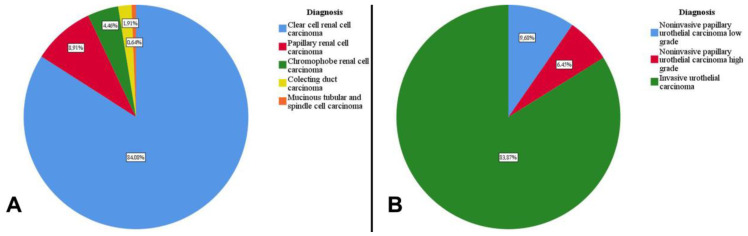
The diagnoses found in the study group. (**A**) Carcinomas originating in the epithelium of the renal tubes. (**B**) Carcinomas of urothelial origin.

In the studied group, the most frequent diagnoses are clear cell renal cell carcinoma, invasive urothelial carcinoma, and papillary renal cell carcinoma (Figure 3).

**Figure 4:**
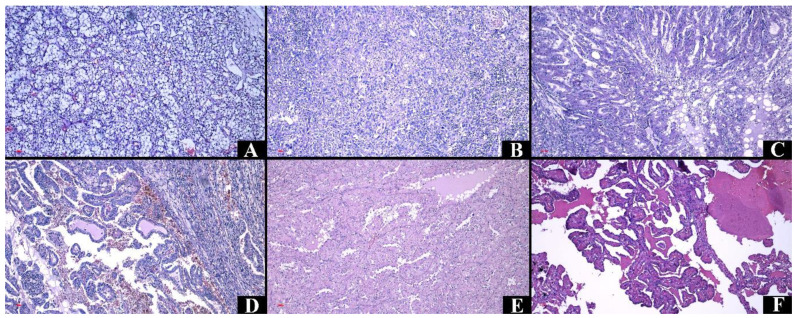
Representative figures of the cases from the study group in hematoxylin–eosin staining, Ob. ×100. (**A**) Clear cell renal cell carcinoma. (**B**) Clear cell renal cell carcinoma of histological grade 4. (**C**) Clear cell renal cell carcinoma, eosinophilic variant. (**D**) Papillary renal cell carcinoma. (**E**) Papillary renal cell carcinoma, oncocytic variant. (**F**) Papillary renal cell carcinoma with inverted polarity.

**Figure 5:**
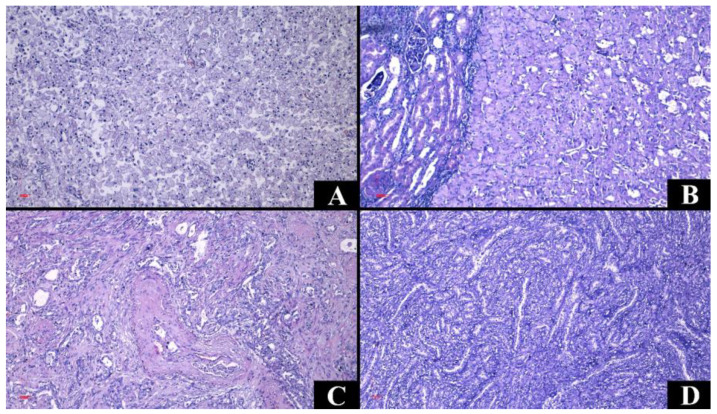
Representative figures of the cases from the study group in hematoxylin–eosin staining, Ob. ×100. (**A**) Chromophobe renal cell carcinoma. (**B**) Chromophobe renal cell carcinoma, eosinophilic variant. (**C**) Carcinoma of collecting ducts, with vascular invasion. (**D**) Mucinous tubular and spindle cell carcinoma.

In RCC, most cases were of clear cell renal cell carcinoma (84.08%), papillary renal carcinoma (8.92%), and chromophobe renal carcinoma (4.46%) (Figure 4 and Figure 5).

**Figure 6:**
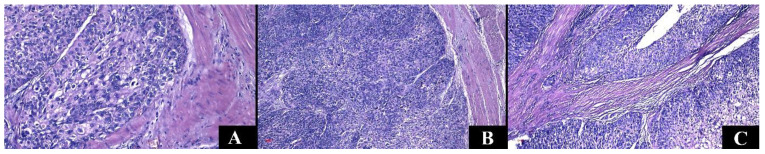
Representative figures of the cases from the study group in Hematoxylin–Eosin staining. (**A**) Noninvasive papillary urothelial carcinoma low grade (Ob. ×200). (**B**) Noninvasive papillary urothelial carcinoma high grade (Ob. ×100). (**C**) Invasive urothelial carcinoma (Ob. ×100).

In the case of tumors of urothelial origin, the most common diagnoses are invasive urothelial carcinoma (83.87%), followed by low-grade (9.68%) and high-grade (6.45%) noninvasive papillary urothelial carcinomas (Figure 6).

In RCC, we observed a higher frequency of hemorrhagic infiltrate, while in UTUC, the aggressive behavior is highlighted by the increased frequency of angiolymphatic and perineural invasions. Regarding the background pathology associated with tumors, we observed a diversity in conditions such as interstitial nephritis associated with RCC or chronic pyelonephritis associated with UTUC (Table 5).

In RCC, the infiltrative aspect is observed with preference in cases of papillary renal carcinoma and in collecting duct carcinomas (*p* = 0.027). The infiltrative aspect of the tumors is associated with their increased axial diameters (*p* < 0.001). Also, this aspect is correlated with the presence of an acute or mixed intratumoral inflammatory infiltrate (*p* = 0.006), with the presence of tumor necrosis (*p* < 0.001), of hemorrhagic infiltrate (*p* = 0.035), with high nuclear grade (*p* = 0.026), and with lymphovascular invasion (*p* < 0.001). From a clinical and paraclinical point of view, the infiltrative aspect is associated with acute renal injury (*p* = 0.023), elevated creatinine levels (*p* = 0.013), and the presence of dysuria (*p* = 0.015). A maximum diameter of ≥6.25 cm has a sensitivity of 71.1% and a specificity of 72.3% for the invasive character (AUC = 0.778, *p* < 0.001). More precisely, the same cut-off for exceeding the renal capsule has a sensitivity of 70.4% and a specificity of 68.5% (AUC = 0.749, *p* < 0.001). For invasion of the pyelocalyceal system, it has a sensitivity of 69.6% and a specificity of 67.2% (AUC = 0.750, *p* < 0.001). Also, a creatinine level of ≥0.91 mg/dL is predictive of invasiveness with a sensitivity of 60.5% and a specificity of 60.5% (AUC = 0.634, *p* = 0.013).

Patients who presented with high blood pressure at admission had a decreased maximum diameter of the tumor (*p* = 0.041). The lesions with increased diameter are associated with exceeding the renal capsule (*p* < 0.001), invasion of the pyelocalyceal system (*p* < 0.001), tumor necrosis (*p* < 0.001), hemorrhagic infiltrate (*p* = 0.010), lymphovascular invasion (*p* < 0.001), and high nuclear grade (*p* < 0.001). A statistically significant difference is observed between tumor diameter and hematuria. Thus, patients with macroscopic hematuria have larger tumors than those with microscopic hematuria or those without hematuria (*p* = 0.035).

The lesions that exceeded the renal capsule are associated with the invasion of the pyelocalyceal system (*p* < 0.001), lymphovascular invasion (*p* = 0.006), and the presence of necrosis (*p* = 0.005). This occurs more frequently in the case of patients from an urban environment (*p* = 0.019). From a paraclinical point of view, exceeding the renal capsule is associated with symptoms such as oligoanuria (*p* = 0.017), dysuria (*p* = 0.036), as well as with the presence of anemia (*p* = 0.031), without correlating with its degree (*p* = 0.641). Exceeding the renal capsule is associated with the death of patients (*p* = 0.049).

The presence of tumor necrosis is associated with intratumoral hemorrhagic infiltrate (*p* < 0.001), pyelocalyceal system invasion (*p* = 0.006), angiolymphatic invasion (*p* = 0.036), and acute or mixed inflammatory infiltrate (*p* = 0.008). Renal weight is higher in the presence of tumor necrosis (*p* < 0.001). A maximum diameter of ≥4.8 cm is predictive of tumor necrosis with a sensitivity of 74.2% and a specificity of 61.8% (AUC = 0.726, *p* < 0.001).

A tumor diameter of ≥5.35 cm shows a sensitivity of 70% and a specificity of 65.5% for a high nuclear grade (AUC = 0.707, *p* < 0.001). Also, a diameter of ≥6.75 cm is associated with the presence of sarcomatoid areas (nuclear grade 4), presenting a sensitivity of 71.4% and specificity of 65.3% (AUC = 0.733, *p* = 0.037). Acute or mixed intratumoral inflammatory infiltrate correlates with angiolymphatic invasion (*p* = 0.036) and high nuclear grade (*p* = 0.033). Precisely, the angiolymphatic invasion is associated with high nuclear grade (*p* < 0.001), invasion of the pyelocalyceal system (*p* = 0.005), exceeding the capsule (*p* = 0.006), staging pT >T3a (*p* < 0.001), and perineural invasion (*p* =0.031). From a clinical point of view, patients with angiolymphatic invasion present with oligoanuria (*p* = 0.034). At a diameter of ≥5.75 cm, the predictability for angiolymphatic invasion increases (sensitivity of 66.7% and specificity of 62%, AUC = 0.693, *p* < 0.001). Angiolymphatic invasion is associated with the death of patients (*p* = 0.046).

Statistically significant differences were observed between diagnoses and perineural invasion; thus, perineural invasion is predominantly observed in cases of chromophobe renal carcinoma and collecting duct carcinoma (*p* = 0.031).

We observed a statistically significant difference between the maximum diameter of the tumors and the pathology of the remaining parenchyma. We noted an increased diameter determined chronic pyelonephritis, followed by hydronephrosis and interstitial nephritis (*p* = 0.006). We observed a statistically significant difference between the background pathology of the kidney and the creatinine levels. Thus, this is increased in hydronephrosis and chronic pyelonephritis (*p* = 0.008).

In UTUC, we observed differences between the diagnosis and the presence of acute renal injury, this being predominantly present in cases of invasive urothelial carcinoma (*p* = 0.018). We observed the same aspect in the case of anemia (*p* = 0.007) and inflammatory syndrome (*p* = 0.018). In the cases of invasive urothelial carcinoma and high-grade papillary noninvasive urothelial carcinoma, we observed associations with macroscopic hematuria (*p* = 0.019).

The presence of necrosis is associated with anemia (*p* = 0.042), and acute renal injury (*p* = 0.022), without associations with creatinine levels (*p* = 0.503), as well as with the infiltrative character (*p* = 0.016). It is also associated with high-grade lesions (*p* = 0.022), lymphovascular invasion (*p* = 0.039), a stage >pT2 (*p* = 0.008), and with the presence of hemorrhagic infiltrate (*p* = 0.008).

The infiltrative aspect is correlated with the presence of systemic inflammatory syndrome (*p* = 0.010) and acute renal injury (*p* = 0.016), without statistically significant associations with serum creatinine level (*p* = 0.093). Intratumoral hemorrhagic infiltrate correlates with the presence of acute renal injury (*p* = 0.020) and with the presence of perineural invasion (*p* = 0.042), without being associated with angiolymphatic invasion (*p* = 0.473). The presence of intratumoral hemorrhagic infiltrate correlates with background renal pathology such as interstitial nephritis (*p* = 0.042).

Lymphovascular invasion is associated with a stage >pT3 (*p* < 0.001), high-grade tumors (*p* = 0.009), incomplete resection (*p* = 0.043), and perineural invasion (*p* = 0.037), while perineural invasion is correlated with the death rate (*p* = 0.044). Advanced stages (pT3 and pT4) are associated with the presence of the inflammatory syndrome (*p* = 0.004). In the case of high-grade tumors, we observed an association with the clinical picture through manifestations such as hematuria (*p* = 0.030) and acute renal injury (*p* = 0.035).

As expected, high-grade tumors have a larger maximum diameter (*p* = 0.025). A diameter of ≥3.75 cm is predictable for a high-grade tumor with a sensitivity of 78.9% and a specificity of 59.3% (AUC = 0.739, *p* = 0.027). Also, lymphovascular invasion is associated with an increased tumor diameter (*p* = 0.011). At a cut-off of ≥4.05 cm, the sensitivity and specificity are increased (86.7% and 69.7%, respectively) for lymphovascular invasion (AUC = 0.765, *p* = 0.012).

### 3.6. Risk Factors

The analysis of risk factors took into account all studied parameters (demographic, clinical, paraclinical, morphometric, macroscopic, and microscopic).

The univariate analysis of the data identifies major risk factors in terms of mortality for renal cell carcinomas: age, exceeding the renal capsule, a tumor that includes all three renal locations, lymphovascular invasion, high nuclear grade (G3 and G4), acute renal injury, and the presence of anemia. In the case of multivariate analysis, only the presence of age and anemia remains a risk factor for the death of patients (Table 6).

In cases of UTUC, the univariate analysis highlights perineural invasion, acute renal injury, the presence of anemia, and hematuria as the main risk factors associated with mortality (Table 7).

## 4. Discussion

Renal cell carcinomas are on an upward trend globally, especially in North America, eastern Europe, and northern Europe. This fact is caused both by screening and early detection programs (in some states having effects in reducing mortality), as well as by exposure to some risk factors. The most incriminated factors associated with the occurrence of these cancers are the chronic use of diuretics and acetaminophen, urinary tract infections, diet, and occupational exposure factors (asbestos, radiation, trichloroethylene, cadmium, arsenic compounds, etc.) [19,20].

 Also, urothelial carcinomas of the upper tract show an upward trend. This is obvious in the case of advanced age and professions such as printers (exposure to azo dyes) and sailors (exposure to asbestos fibers from ship components). Regarding factors such as gender, race, and geographical location, the data are not standardized, as there are no clear records [21]. Even if these tumors are rare, representing less than 10% of renal tumors, in our study, we managed to identify a percentage of over 15% [2,7].

In the present study, both types of cancer had an undulating evolution, with upward trends followed by a decreasing phase, finding no explanation for this phenomenon.

Two constantly analyzed risk factors in the development of cancers are associated with patients’ habits and consist of smoking and alcohol consumption. In RCCs, smoking induces renal dysfunction through nephrotoxic effects, hemodynamic changes, endothelial dysfunction, and oxidative stress. Aromatic polycyclic hydrocarbons can cause gene mutations, especially in the p53 gene. Nicotine is associated with the alteration of the endothelial growth factor pathway. Smoking has the effect of promoting inflammation by reducing the immune function of T lymphocytes and reducing the activation of natural killer cells. All these injuries can induce DNA damage, accelerate cell turnover, and increase cancer growth and progression [22]. The most recent studies have observed that smokers have twice the risk of developing cancer compared to non-smokers, and former smokers have a 20% greater risk than non-smokers. A dose-dependent relationship was also observed. Thus, the relative risks are 1.18 for 5 cigarettes per day, 1.36 for 10 cigarettes per day, 1.61 for 20 cigarettes per day, and 1.72 for 30 cigarettes per day [14,23]. The study by Gansler et al. noted that compared to non-smokers, smokers are less likely to develop chromophobe renal cell carcinoma, unlike the other histomorphologies of renal cancer [24]. Similarly, in the case of UTUC, smoking is associated with a higher risk of advanced disease, lymph node metastases, recurrences, intravesical recurrences, as well as an increased cancer-specific mortality rate [25,26].

In the case of alcohol consumption, studies have shown a protective effect in RCCs, while in UTUC, alcohol is a risk factor [27,28]. In RCC, moderate alcohol consumption (24–60 g of alcohol per day) has an odds ratio of 0.67 compared to those who do not consume alcohol [27]. Another study indicates that there is a dose-dependent relationship. Thus, for every 5 g per day, the risk decreases by 5% in the case of men and by 9% in the case of women [29]. The possible responsible mechanisms are represented by the diuretic effect that reduces arterial hypertension, as well as by phenolic antioxidant compounds that promote apoptosis and reduce oxidative stress, lipid peroxidation, and cell proliferation [23]. In UTUC, the effect of alcohol is negative, increasing the risk of developing cancer by 1.2–1.5 times more than in the case of those who do not consume alcohol. The threshold at which alcohol becomes a risk factor is >15 g per day. The pathogenic mechanism is represented by the increased presence of acetaldehyde detected in the urine of patients [28].

In addition to risk factors, hereditary syndromes are also identified in the etiology of renal cell cancers (approximately 2% of cases). The most common are found in von Hippel–Lindau disease (VHL gene causing clear cell renal cell carcinoma), BRCA1 gene protein mutation (BAP1 gene causing clear cell renal cell carcinoma), papillary hereditary renal carcinoma (MET gene with production of papillary renal carcinoma type 1), hereditary leiomyomatosis (FH gene with production of papillary renal carcinoma type 2). Among the genes involved in the occurrence of hereditary clear cell renal cell carcinomas, it is worth mentioning ARID1A, KDM5C, PBRM1, SDH, and STED2, and in the occurrence of hereditary non-clear cell carcinomas the genes, FLCN (Birt–Hogg–Dube disease), PTEN (Cowden syndrome), and TSC (tuberous sclerosis) [30,31,32]. In our study, after consulting the observation sheets, we did not notice any family syndromes in the batch.

The most common mechanism involved in the pathogenesis of RCC is represented by the activation of the PI3K/AKT/mTOR pathway. The overexpression of growth factors (EGF, IGF, and VEGF) leads to the activation of RTK, which through signaling pathways (RAS/MEK/ERK) leads to the production of hypoxia-inducible factor (HIF-α). This can lead to mutations at the level of the AKT/mTOR complex. Likewise, mTOR hyperactivity can also be due to low expressions of TSC1/2, epigenetic suppression of the PTEN gene, or inactivation of the VHL gene [33,34]. In the case of papillary renal carcinomas, it has been observed that acute kidney injury can also play a role in carcinogenesis. This promotes the development of cancer through the adenoma–carcinoma sequence or through the overexpression of NOTCH1 in cases of papillary renal carcinoma type 2 [35].

In the etiology of UTUC, associations with Lynch syndrome were found, through mutations of DNA mismatch repair genes, or hEPCAM genes. Another important etiopathogenic element is represented by Balkan endemic nephropathy. This is a tubulointerstitial disease, which leads to the appearance of cancer over time, produced by the phytotoxin aristolochic acid found in the plants from which homemade bread is produced in areas of southeastern Europe [31,36]. The acid forms an aristolactam–DNA complex with the DNA that ends TP53 gene mutations [37]. We do not have any information that affirms or denies the use of aristolochic acid in any form in the studied batch. Pathogenic pathways are similar to urothelial carcinomas of the urinary bladder, consisting of a multistep process [37,38]. Unlike bladder carcinomas, the most affected genes are FGFR3, HRAS, and KMT2D. Affecting genes frequently involved in bladder urothelial carcinomas (TP53, RB1, ERBB2, and KDM6A) are less common in UTUC and are associated with the risk of intravesical recurrence [37].

In RCCs, age represents a risk factor involved in the increased incidence of these tumors. But this is not associated with the death rate. Studies have noted an increased frequency between the sixth and eighth decades of life, with a peak incidence between 60 and 70 years [13]. In the present study, we noted the same aspect of the distribution of renal carcinoma cases, with a median age of 64 years. We found that an age ≥64.5 years has increased predictability for death.

The same upward trend in age correlated with incidence is also observed in the case of UTUC. Most cases are identified in people over 60 years old, with a peak over 75 years old [21,39]. In our group, the peak frequency was 73 years, lower than in the literature.

In RCC, the data regarding the distribution according to sex are contradictory. Some studies have indicated an increased prevalence in males, others in females. This aspect may be due to histopathological subtypes (chromophobe renal carcinoma and papillary renal carcinoma have an increased frequency in females), occupational factors, comorbidities, or lifestyle [40,41]. The present study supports the aspects that men are most frequently affected, in conjunction with the increased frequency of clear cell renal cell carcinomas.

In UTUC, a higher frequency has been observed for males (59.9–68.4%), an aspect also found in the present study (61.54%). However, survival was not markedly influenced [42]. An aspect noted by Deuker M et al. was the differences in the distribution of UTUC metastases according to sex. They observed that bone metastases occur more frequently in men, and liver metastases in women [43]. In our study, we identified only two cases of bone metastases, both in males.

More than half of all RCCs are diagnosed incidentally; the classic triad (flank pain, hematuria, and palpable abdominal mass) is rarely present. As a rule, paraneoplastic syndromes such as hypercalcemia, erythrocytosis, Stauffer syndrome, or unexplained fever are associated [44]. Paraclinical tests such as serum creatinine, blood count with an emphasis on the number of leukocytes and platelets, lactate dehydrogenase, C-reactive protein, hemoglobin, and serum-corrected calcium also contribute to the clinical examinations. Paraclinical tests are useful in the International Metastatic Database Consortium (IMDC) scoring system for prognosis in advanced stages [44,45].

On the other hand, patients with urothelial carcinomas are symptomatic in two-thirds of the cases. Most often, they present with macroscopic or microscopic hematuria, less often with pain in the flank or signs of hydronephrosis [7]. In endemic areas, cases of “Blackfoot disease” have been reported, especially in the elderly [46].

In the present study, we observed that patients with RCCs presented with colicky flank pain, macro- and microscopic hematuria, and dysuria. In the case of UTUC patients, the dominant sign was macroscopic hematuria, followed by colicky flank pain. In the case of UTUC, hematuria was a negative prognostic factor regarding survival (*p* = 0.038).

The most common comorbidities are represented by hypertension, diabetes, obesity, and acute kidney injury. Hypertension is recognized as a risk factor associated with renal cell carcinomas. Studies have shown that a 10 mmHg increase in systolic blood pressure increases the risk by 5%, and a 10 mmHg increase in diastolic blood pressure increases the risk by 7% [47,48]. Recently, it was found that antihypertensive drugs are involved in the occurrence of these cancers. In this sense, a linear relationship between the duration of medication use and a risk that increases by 2% per year was observed, as well as in the case of the use of two or more classes of antihypertensives (HR = 1.80) [49,50]. In the case of UTUC, patients with arterial hypertension developed high-grade tumors and had lower overall survival [51,52]. In the group of patients we studied, we observed the association of arterial hypertension with other comorbidities, while survival was not affected.

The role of diabetes in the occurrence of cancers is still being studied, as it is involved in numerous neoplastic diseases. In RCCs, its importance is unclear. Most studies have shown an association between the occurrence of renal cell carcinoma and the female sex, without association with the male sex [53]. In the present study, diabetes was more frequently encountered in the case of men (66.67%) with renal cell carcinomas, without statistical significance (*p* = 0.195). The main mechanisms by which diabetes intervenes are represented by the pro-inflammatory effect, insulin resistance, and excessive compensatory insulin production. They overlap with or are synergistic with carcinogenic mechanisms [54]. In UTUC, the presence of diabetes did not have a significant impact on patient survival, an aspect also identified by us. Instead, its presence was associated with an increased risk of intravesical recurrence [55]. During the follow-up period, no patient developed recurrences.

Regardless of the quantification (body mass index, waist circumference, hip circumference, or percentage of body fat), obesity increases the risk of RCC (35–76%) [56]. The main mechanism involved consists of renal hypoxia and stimulation of the vascular endothelial growth factor pathway. This is associated with renal hyperfiltration, which increases exposure to nephrotoxins [47]. On the other hand, a study conducted by van de Pol et al. noted that an increased body mass index increases the risk for clear cell renal cell carcinomas and decreases that of papillary renal carcinomas [57]. In the present study, we observed an increased frequency of obesity, calculated based on the body mass index, in clear cell renal cell carcinomas (19.70% of them), in contrast to papillary renal carcinomas (7.14% of them), without statistical significance (*p* = 0.840). On the other hand, obesity is not associated with the occurrence of UTUC, but it is associated with a low outcome, an aspect not identified in our study [58]. Instead, more reliable indicators for the association with the occurrence, as well as with the prognosis are represented by body composition parameters that include the association of muscle mass and adipose tissue or the metabolic syndrome that includes obesity, hypertension, dyslipidemia, and elevated fasting blood glucose [58,59].

An increase in serum creatinine of ≥50% in 7 days or ≥0.3 mg/dL in 2 days defines acute renal injury. An episode is a risk for the development of RCC, and if the episode occurs after a partial nephrectomy after kidney cancer, the risk of recurrence will increase [60]. In our study, we observed that elevated preoperative creatinine was associated with the death rate. Moreover, a threshold of ≥0.95 mg/dL is predictive of survival. Thus, acute renal injury represents a negative prognostic factor (HR = 2.734, *p* = 0.010). Patients who associate acute kidney injury with obesity and diabetes have an increased risk of damage to the proximal tubules, which accentuates cortical damage with the possible onset of renal cell carcinoma [61]. Two interesting aspects arise from the specific topographical damage. Thus, lesions of the proximal tubules in the S1 and S2 segments potentiate the development of clear cell renal cell carcinoma, and damage to the S3 segments increases the risk of developing papillary renal carcinomas through the adenoma–carcinoma axis [35,62]. Damage to the collecting ducts leads to the development of chromophobe carcinoma, or carcinoma of the collecting ducts [62]. A meta-analysis conducted by Mori et al. noted that creatinine levels in UTUC were not statistically significantly associated with cancer-specific survival [63]. Instead, Liu et al. noted that there is a close association between elevated levels of serum creatinine and the presence of oxidative stress. Because of this association, they concluded that serum creatinine levels are an independent risk factor for mortality [64]. This aspect was not identified in the present study. Other researchers in the field have observed that a higher fidelity in the disease-specific and recurrence-free survival rate is represented by the eGFR decrease <60 mL/min/1.73 m^2^ [65].

Two aspects that can be identified paraclinically, the presence of anemia and systemic inflammatory syndrome, play an essential role in the patient’s evolution. In RCCs, studies are contradictory regarding the association between anemia and cancer-specific survival. However, the effects they have cannot be neglected. Among the causes of preoperative anemia that indicate increased aggressiveness are marrow infiltration, hemolysis, and red cell aplasia. A frequently encountered cause is represented by the association with hematuria [66]. In our study, anemia was associated with hematuria only in the case of renal cell carcinomas (*p* = 0.009). The presence of anemic syndrome was also a negative prognostic factor regarding survival (HR = 2.875, *p* = 0.007). An aspect that should not be neglected, which contributes to the low survival of patients, consists of the low threshold of patients requiring perioperative blood transfusion [66]. The most important aspect consists of the main consequence of anemia, hypoxia. The increased expressions of HIF1α and HIF2α were associated with an unfavorable prognosis in patients with renal cell carcinoma [66,67]. The same hypoxic mechanism is also found in the case of anemia from UTUC. In these situations, anemia is a negative risk factor for cancer-specific survival and overall survival [68,69]. In addition, hypoxia causes increased levels of vascular endothelial growth factor (VEGF). These are responsible for a decrease in the incidence of recurrence-free survival as well as for the progression of bladder cancer [68,70]. In the group of patients with UTUC, anemia was not associated with patient survival, but its cause (hematuria) was correlated with their death.

Cancer-related inflammation is intensively studied and refers to the inflammatory cell infiltrate and the activated stroma. An aspect that tends to be neglected is the systemic inflammatory response. It includes the quantification of blood elements and the levels of hemoglobin and albumin [71]. Over time, systems have been identified to indicate the prognosis depending on the ratios of the inflammatory elements in the peripheral blood: neutrophil-to-lymphocyte ratio (NLR), platelet-to-lymphocyte ratio (PLR), and monocyte-to-lymphocyte ratio (MLR) [72,73]. The systemic inflammatory response index (SIRI) is a stronger predictor than the others, regarding overall survival and cancer-specific survival, in advanced renal cell carcinomas. In low-grade renal cell carcinomas or early stages, this system has a strong prognostic value for recurrence or metastasis [72]. In UTUC, the three systems (NLR, PLR, and MLR) are associated with recurrence, decreased overall survival, and decreased cancer-specific survival. Similar to RCCs, a new systemic immune-inflammation index (SII) is associated with oncological outcome, total survival, and cancer-specific survival in UTUCs. The highest prognostic accuracy is identified in the association of SII with MLR [74,75]. In both types of cancer, systemic inflammatory syndrome was associated with acute renal injury, implicitly with increased levels of serum creatinine.

The presence of bilateral renal cell carcinomas is frequently identified in hereditary syndromes (synchronous or metachronous tumors), while in sporadic cases, multifocality is more frequently encountered [76]. In the group of patients with RCC, none were documented with hereditary syndromes or with bilateral or multiple carcinomas. Until now, studies have not found clear evidence of differences in overall survival in these situations [76]. Regarding laterality, Guo S et al. observed that right kidney lesions are associated with early stage and better survival [77]. In our case, the left kidney was the most affected, not being associated with early or advanced stages (*p* = 0.750) or with survival (*p* = 0.580). Also, laterality is a prognostic factor only in tumors ≥10 cm [77]. In the present study, we observed that a large tumor that manages to occupy all renal thirds represents an important risk factor associated with death. In urothelial carcinomas developed at the renal level, the location does not influence survival, an aspect noted by us as well [78].

In addition to the characteristic elements of macroscopy and microscopy, as evidenced in Table 8, the pathologist must point out certain essential elements. The initial stages (T1–T2) of RCCs are characterized by the size of the lesions. Advanced stages (T3–T4) involve a tumor that invades segmental branches of the renal vein, perirenal fat, renal sinus fat, vena cava below or above the diaphragm, beyond Gerota’s fascia, or the ipsilateral adrenal gland [79]. These elements have major implications; this fact is supported by the present study. Thus, in the case of tumors that exceed the renal capsule, we identified a risk factor associated with death (HR = 2.504, *p* = 0.040). In UTUC, the most important aspects consist of highlighting the invasion, whether it is present or not. If it is present, its depth matters: subepithelial connective tissue, muscle, perirenal fat, or renal parenchyma, as well as the adjacent organs by invasion from opening to opening [80].

Tumor diameter has implications, especially for the metastatic capacity of RCC. Chromophobe carcinoma has a low risk of metastasis, while papillary and clear cell carcinomas have a progressively increasing risk starting at 3 cm, up to a plateau phase at 12 cm [87]. In the present batch, only two clear cell renal cell carcinomas with diameters of 6 cm and 12.4 cm presented metastases at the time of diagnosis, both at the lung level. In urothelial carcinomas of the upper urinary tract, it is considered that a large tumor is associated with the invasion of the muscular tunic and implicitly has a negative prognosis. Following these aspects, a diameter of 2 cm was agreed upon to stratify the recurrences of these tumors [88,89]. In the present group, the urothelial carcinomas that presented bone metastases had diameters of 5.7 cm and 7 cm.

Tumor necrosis is a consequence of exceeding the oxygen requirement, leading to hypoxia and cell death. In clear cell renal cell carcinomas, its presence has been associated with metastases and recurrence [90]. Also, in the case of chromophobe renal carcinomas, the presence of tumor necrosis was associated with the risk of metastasis as well as a lower specific cancer survival rate [91]. Aspects that we did not find. An element that should not be neglected is the presence of dirty necrosis. This is composed of the area of tumor necrosis with which neutrophilic infiltrates are associated. Its presence is associated with the phenomenon of systemic inflammation [92]. We identified an association between necrosis and acute intratumoral inflammatory infiltrate (*p* = 0.008) but without a correlation between them and the systemic inflammatory syndrome (*p* = 0.872). In the case of UTUC, a necrosis threshold of over 10% is associated with metastases and cancer deaths [93]. In our study, the presence of necrosis was not associated with the death of patients (*p* = 0.461). Instead, it was associated with elements known in the literature as predictive factors of mortality, such as high grade, invasive character, or angiolymphatic invasion.

The presence of the hemorrhagic infiltrate is caused by spontaneous rupture, the mechanism of which is not fully elucidated. There are hypotheses according to which this fact is caused by the invasion of the capsular tumor, of the vascular structures, or by the tension exerted by the rapid growth of the tumor mass [94]. In both types of cancer developed at the kidney level, the incidence is low due to non-reporting [94,95]. In this study, the intratumoral hemorrhagic infiltrate was quantified, observing a higher frequency of it in RCC. We assume that the presence is due to the tension exerted by the rapid growth of renal tumors because we identified associations between hemorrhage and the increased diameter of the tumors (*p* = 0.010). In patients with UTUC, hemorrhage was less frequent and correlated with tumor necrosis (*p* = 0.008) and perineural invasion (*p* = 0.042).

Recent studies have focused on methods for identifying and quantifying intratumoral inflammatory infiltrates. Some research has demonstrated its effectiveness as a predictor of the therapeutic strategy and prognosis of patients. For example, a low number of lymphocytes is associated with the suppression of the immune system and implicitly with a poor prognosis. Other essential elements of the inflammatory infiltrate are represented by neutrophils. They are called at the tumor level using cytokine secretions by the tumor cells. Neutrophils contribute to tumor progression by diverting anti-tumor immunity by inducing angiogenic and lymphangiogenic and promoting cell proliferation phenomena [96]. In the studied renal cell carcinomas, we observed the presence of an inflammatory infiltrate containing neutrophils (acute or mixed) associated with the infiltrative character (*p* = 0.006). This aspect supports the role of neutrophils in promoting cell proliferation and tumor aggressiveness. The most studied inflammatory population is represented by lymphocytes, mainly CD4+ and CD8+. These are also potential therapeutic targets for certain cancers that bring clinical improvement [97]. In addition, in UTUC, the increased stromal lymphocytic tumor infiltrate brings improvements in locally advanced cases [98].

The histological grade of RCCs is represented by the modified ISUP system [99]. Regarding it, the studies are contradictory regarding the prognosis. Some studies consider only grade 4 as a risk factor; others have observed differences between low grades (1 and 2) and high grades (3 and 4); and in clear cell renal cell carcinoma, differences have been observed between grades 2, 3, and 4 [15,100,101]. We observed statistically significant differences in terms of patient survival only between high-grade (3 and 4) and low-grade (1 and 2). Death is associated with high-grade tumors (*p* = 0.042), high grade also being a negative prognostic factor (HR = 2.172, *p* = 0.039) in renal cell carcinomas, not only in clear cell renal cell carcinomas. In the case of urothelial tumors, some associations still recommend the use of both classification systems (two levels and three levels). The justification for using the two-level system derives from the pathogenic process. Low-grade carcinomas show FGFR3 alterations in 80% of cases, have a high recurrence rate, and have non-aggressive behavior. The high-grade ones frequently have TP53 alterations, aggressive behavior, and an increased risk of invasive progression [102]. We observed an increased frequency of deaths among patients with high-grade urothelial tumors (57.89% vs. 25%) but without statistical significance (*p* = 0.073).

Studies have shown that renal cell carcinomas often metastasize through hematogenous microvesicles. The most affected organs are the adrenal glands (7–23%), the brain (2–17%), the pancreas (2%), the urinary bladder, and bones [103]. Of all these, the Groupe Francais d’Immunotherapie noted that liver and bone dissemination, along with other aspects, are predictive factors for poor overall survival [104]. Even if we detected angiolymphatic invasion more frequently in cases of UTUC, in RCC, this represents an important risk factor regarding death (HR = 2.444, *p* = 0.029).

In cases of UTUC, the most frequent modes of dissemination are lymphatic and vascular. The most involved organs are the lungs (55%), distant lymph nodes (37%), bones (32%), and the liver (20%) [105]. Unlike ureteral urothelial carcinomas, those in the renal pelvis have a better evolution. This is explained by the fact that the ureteral adventitia is thin and rich in lymphatic and blood vessels; they create an opportune terrain for metastasis, while the renal parenchyma and perirenal adipose tissue act as a barrier against dissemination [106]. Perineural dissemination is underdiagnosed and often not reported [107,108]. However, numerous cases have been observed in which, along the T10-L2 spinal nerves, as well as through vegetative threads, the renal tumor can spread to the thoracic, duodenal, pancreatic, or intradural level [107]. The study of Lin TW et al. identified that in UTUC, perineural invasion is a prognostic factor for progression-free survival (HR = 1.724), cancer-specific survival (HR = 2.544), and overall survival (HR = 1.779). On the other hand, in urothelial carcinomas of the urinary bladder, perineural invasion is not a predictive marker for adverse outcomes [108]. In our study, we observed a higher frequency of dissemination of urothelial tumors compared to RCC. Moreover, like in Lin TW et al., perineural invasion represented a risk factor regarding the death of patients (HR = 7.500, *p* = 0.029).

In performed nephrectomies, the non-neoplastic renal pathology is not recognized for various reasons; most of the time, it is generically reported in the form of chronic renal disease. Tubulointerstitial disorders are more frequent in RCC, representing a consequence of glomerular destruction [109]. On the other hand, UTUC is associated with hydronephrosis. Its presence is associated with a high stage at the time of diagnosis, a higher grade, and low overall survival [110,111]. Also, the presence of hydronephrosis may involve the extension of tumor cells through all layers of the urothelium, favoring nodal or visceral dissemination. This aspect is due to the increased intraluminal pressure that can cause counterflow in the blood and lymphatic vessels [111]. In the present study, we observed an association between the presence of hydronephrosis and chronic pyelonephritis in cases of UTUC (*p* = 0.012), but without any correlation with angiolymphatic (*p* = 0.789) or perineural (*p* = 0.776) dissemination.

The management of renal cell tumors is based on surgical intervention. This can be achieved through open surgery or minimally invasive techniques (laparoscopic or robotically assisted). Alternative therapeutic strategies consist of active surveillance and thermal ablation (Table 9) [84,112]. In our study, renal cell carcinomas had a better surgical resection rate than urothelial carcinomas, as verified by complementary imaging examinations. In cases of metastasized renal cell carcinomas, strategies including targeted therapies and immunotherapeutic agents have been approved. The most commonly used targeted therapies are sorafenib, sunitinib, pazopanib, cabozantinib, lenvatinib, and axitinib. In the case of immunotherapy, interferon-α is used with high doses of interleukin 2 (IL-2) or immune checkpoint inhibitors (atezolizumab, avelumab, pembrolizumab, and nivolumab) [84].

UTUC management consists of the division into two risk classes. The criteria for low-risk tumors are size under 2 cm, unifocal lesion, and low-grade or noninvasive lesion, and the high-risk criteria are the presence of hydronephrosis, multifocal lesion, tumor over 2 cm, and high-grade or invasive lesion [5]. The standard treatment for low-risk tumors consists of kidney-sparing surgery through segmental ureterectomy, ureteroscopy, or percutaneous intervention [115]. Afterward, topical instillation therapy with mitomycin B or bacillus Calmette–Guerin can be used to reduce the rate of recurrence and progression [116]. For high-grade tumors, radical renoureterectomy with a bladder cuff is performed through an open surgical approach or minimally invasive techniques (laparoscopic or robotically assisted). Some guidelines also recommend regional lymph node dissection. In this case, topical intravesical instillation can be used to reduce the rate of bladder recurrence [116,117]. In advanced cases, systemic therapy is used (e.g., gemcitabine and cisplatin or methotrexate, vinblastine, Adriamycin, and cisplatin), radiotherapy, or immunotherapy (pembrolizumab, nivolumab, durvalumab, and avelumab) [117].

The role of lymph node dissection in the management of renal carcinomas (renal cell carcinoma and UTUC) remains controversial. Some studies and trials have identified a beneficial role (complete staging, prognosis, and survival), while others have not identified improvement in prognosis or survival [118,119]. For this reason, there is no standardized model of dissection. Still, the excision of the hilar, precaval, and interaorticaval nodes for the right kidney and the hilar, paraaortic, and interaorticaval nodes for the left kidney is recommended [119,120].

It can be said that both types of tumors that can develop at the level of the kidney are found in elderly people, have a similar clinical picture, and present similar risk factors and prognostic factors that sometimes overlap [121]. Even if UTUC is accompanied by several comorbidities (advanced age, lower renal function, and low-performance status), renal function is not as significantly influenced postoperatively, as is the case in renal cell carcinoma [122].

The limitations of this study are its retrospective nature, the absence of data on the patients’ habits (smoking, alcohol, etc.), and the low diversity in some tumor subtypes. A strong point is represented by the correlation of renal morphometry with demographic, clinical, paraclinical, and histopathological aspects. Other strengths of the present study consist of the identification of aspects that improve the specialized literature and open the way for future research perspectives. In the case of UTUC, we identified prognostic factors associated with death (anemia, hematuria, acute renal injury, and perineural invasion). Also, we highlighted certain values of the tumor diameter that are predictable for tumor grade or lymphovascular invasion. In renal cell carcinomas, we managed to identify certain cut-off values of the tumor diameter that correlate with aggressiveness (exceeding the renal capsule, invasion of the pyelocalyceal system, the presence of necrosis, angiolymphatic invasion, nuclear grade, and the presence of sarcomatoid areas), as well as prognostic factors of death (age, exceeding the renal capsule, injury involving the entire kidney, lymphovascular invasion, high nuclear grade, acute renal injury, and anemia). In addition, based on certain values of creatinine, a prediction can be made regarding the invasiveness and the risk of death. Even if they are two completely different tumor entities, they have common clinical–paraclinical characteristics, with small variations in their frequencies. Comparatively, we observed a higher death rate and a higher tendency of dissemination in the case of UTUC. Even if anemia and acute renal injury (implicitly elevated preoperative creatinine levels) were more frequently encountered in UTUC, these parameters had a much greater impact on renal cell carcinomas, both in predicting death and in patient prognosis. Although this study is a long one, it manages to draw up a complete picture of the two pathologies, which it analyzes both individually and in comparison with each other and with the specialized literature.

## 5. Conclusions

Following this study, we managed to identify, both comparatively and individually, the main parameters for predicting the aggressiveness and the risk factors associated with the death of the two tumor entities. Also, by reviewing the specialized literature, we were able to observe common elements with the present study, especially in the case of renal cell carcinomas (aspects of the advanced stage: exceeding the renal capsule, invasion of the pyelocalyceal system; increased histological grade: the presence of sarcomatoid areas and angiolymphatic invasion).

The particular aspects of the identified renal cell cancers are represented by the cut-off values of the tumor diameters correlating with the biological character, as well as the identification of some paraclinical parameters that are associated with the death rate. In urothelial carcinomas of the upper tract, the identified peculiarities consist of identifying hematuria, anemia, acute kidney injury, and perineural invasion as risk factors predicting mortality.

Even if they are two completely different tumoral entities, renal localization, the clinical picture, and biochemical tests cannot make a clear distinction between them, but they can establish vital prognostic elements for patients.

## Figures and Tables

**Table 1:** Demographic aspects of the study group.

	Renal Cell Carcinomas	Upper Tract Urothelial Carcinomas	*p*-Value
Age (years)			<0.001
Median (percentiles 25–75)	64 (56–70)	73 (63–81)	
Min–max	28–81	45–87	
Decade (n, %)	7, 38.85%	9, 32.26%	<0.001
Gender			0.842
Female	42.68%	38.71%	
Male	57.32%	61.29%	
Environment			0.167
Rural	47.13%	32.26%	
Urban	52.87%	67.74%	
Deaths	24.85%	45.16%	0.029

**Table 2:** Clinical and paraclinical aspects.

	Renal Cell Carcinomas	Upper Tract Urothelial Carcinomas	*p*-Value
Hematuria			0.004
Absence	22.29%	3.23%	
Microscopic	36.94%	25.81%	
Macroscopic	40.77%	70.97%	
Flank pain	71.34%	48.39%	0.020
Oligoanuria	11.46%	25.81%	0.046
Dysuria	47.77%	32.26%	0.120
High blood pressure	59.23%	70.97%	0.313
Diabetes mellitus	24.84%	25.81%	0.910
Obesity	17.83%	25.81%	0.321
Acute kidney injury	27.39%	64.52%	<0.001
Serum creatinine (mg/dL)			<0.001
Median (percentiles 25–75)	0.88 (0.73–1.26)	1.34 (1.02–1.72)	
Min–max	0.29–12.62	0.69–26.16	
Anemia	26.75%	70.97%	<0.001
Mild	52.38%	40.91%	
Moderate	33.33%	40.91%	0.721
Severe	14.29%	18.18%	
Systemic inflammatory syndrome	57.96%	64.52%	0.553

**Table 3:** Aspects of renal morphometry and tumor topography.

	Renal Cell Carcinomas	Upper Tract Urothelial Carcinomas	*p*-Value
Weight (g)			0.065
Median (percentiles 25–75)	339 (250–468)	310 (230–350)	
Min–max	120–1665	145–547	
Length (cm)			0.069
Median (percentiles 25–75)	12 (11–14)	12 (11–13.50)	
Min–max	7.5–21	8.5–18	
Kidney			0.845
Left	51.59%	54.84%	
Right	48.41%	45.16%	
Localization			0.065
Superior	40.13%	25.81%	
Middle third/pelvis	28.66%	45.16%	
Inferior	28.03%	19.35%	
All	3.18%	9.68%	

**Table 4:** Macroscopic aspects.

	Renal Cell Carcinomas	Upper Tract Urothelial Carcinomas	*p*-Value
Pseudoencapsulation	91.08%	0%	<0.001
Cystic spaces	52.87%	6.45%	<0.001
Infiltrative appearance	24.20%	87.10%	<0.001
Exceeding the renal capsule	17.19%	6.45%	0.176
Invasion of the pyelocalyceal system	14.65%	-	-
Maximum tumor diameter (cm)			
Median (percentiles 25–75)	5.5 (3.95–7.5)	4.5 (3.5–5.8)	0.025
Min–max	0.9–19	0.7–8	

**Table 5:** Histopathological aspects.

	Renal Cell Carcinomas	Upper Tract Urothelial Carcinomas	*p*-Value
Tumor necrosis	56.69%	61.29%	0.694
Hemorrhagic infiltrate	72.61%	38.71%	0.001
Intratumoral inflammatory infiltrate			
Acute	2.55%	0%	0.41
Mixed	8.92%	16.13%	
Chronic	88.53%	83.87%	
Angiolymphatic invasion	22.92%	48.39%	0.007
Perineural invasion	7.64%	29.03%	0.002
Histological grade	ISUP 2—41.40%		
Low grade (1 and 2)	55.41%	38.71%	-
High grade (3 and 4)	44.59%	61.29%	0.115
pT	3a—31.21%	3—64.52%	-
Metastasis (n)	2—lung	2—bone	-
Complete resection	98.73%	87.10%	0.007
Adjacent renal parenchyma			0.001
Chronic pyelonephritis	35.67%	70.97%	
Normal	31.85%	3.23%	
Interstitial nephritis	25.48%	19.35%	
Hydronephrosis	1.27%	6.45%	

**Table 6:** Univariate and multivariate analysis of risk factors in renal cell carcinomas.

	Univariate Analysis			Multivariate Analysis		
Parameter	Hazard Risk	*p*-Value	CI95%	Hazard Risk	*p*-Value	CI95%
Age	1.073	0.002	1.027–1.121	1.084	0.002	1.031–1.139
Exceeding the renal capsule	2.504	0.040	1.045–6.003	1.255	0.693	0.407–3.871
The lesion throughout the kidney	10.000	0.046	1.045–95.683	5.110	0.238	0.341–76.590
ILV	2.444	0.029	1.096–5.448	1.635	0.344	0.591–4.519
High grade	2.172	0.039	1.039–4.537	2.242	0.076	0.919–5.469
Acute kidney injury	2.734	0.010	1.268–5.895	2.023	0.126	0.820–4.990
Anemia	2.875	0.007	1.329–6.219	2.838	0.028	1.122–7.179

**Table 7:** Univariate analysis of risk factors in UTUC.

	Univariate Analysis		
Parameter	Hazard Risk	*p*-Value	CI95%
Perineural invasion	7.500	0.029	1.228–45.807
Acute kidney injury	6.750	0.035	1.145–39.796
Anemia	11.556	0.033	1.223–109.185
Hematuria	10.448	0.038	1.138–95.926

**Table 8:** Macroscopic and microscopic aspects of the main carcinomas developed at the renal level [81,82,83,84,85,86].

Tumor Type	Variants	Gross Aspect	Origin	Histologic Features
Clear cell renal cell carcinoma	Classic Eosinophilic	Pseudoencapsulated Golden yellow Necrosis Hemorrhage	Tubular epithelium Proximal nephron	Nests and sheets of cells with clear cytoplasm.
Papillary renal cell carcinoma	Type 1 Type 2	Often pseudoencapsulated Solid or cystic Whitish Necrosis Hemorrhage	Tubular epithelium Distal nephron	Thin or thick papillae lined by uni- or pseudostratified cuboidal epithelium, foamy macrophages, and psammomatous bodies.
Chromophobe renal cell carcinoma	Classic Eosinophilic	Well defined Gray to tan-brown Central scar	Intercalated cells of the distal tubules Distal nephron	Cells with prominent membrane and pale/eosinophilic cytoplasm.
Carcinoma of collecting ducts		Partially cystic Grayish-white	Collector tubes	Tubulopapillary architecture, hobnail cells, mucinous material, desmoplastic stroma.
Noninvasive urothelial carcinoma	Papillary: Low grade High grade	Flat or exophytic lesion	Urothelium	Varying degrees of cytoarchitectural atypia (fusion of papillae, disorganized tumor cells); cells with moderate or increased pleomorphism and mitotic activity.
Invasive urothelial carcinoma	Micropapillary Nested Large nested Tubular and microcystic Plasmacytoid Sarcomatoid Lipid-rich Lymphoepithelioma-like Clear cell With differentiation: Squamous Glandular Trophoblastic Mullerian.	Sessile, polypoid, nodular, or ulcerative lesion	Urothelium	Urothelial cells with high-grade atypia can associate divergent differentiation. Various architectures: papillary, micropapillary, nested, or tubular.

**Table 9:** Therapeutic management of localized renal cell carcinomas [84,112,113,114].

Therapeutic Management		Recommendations	Risks
Active surveillance		Tumors ≤ 4 cm in the elderly Severe comorbidities Short life expectancy Absent/atrophic contralateral kidney Reduced baseline eGFR Baseline proteinuria	Cumulative risk of radiation
Ablative techniques	Cryotherapy Radiofrequency ablation	Advanced age Increased number of comorbidities Risk for general anesthesia Single kidney Impossibility of performing major procedures Absent/atrophic contralateral kidney Reduced baseline eGFR Baseline proteinuria	Renal bleeding Formation of abscesses Touching adjacent organs
Surgical techniques	Partial nephrectomy Nephron preservation surgery	Younger age Genetic/familial syndromes T1 and T2 tumors except those whose location makes resection impossible Absent/atrophic contralateral kidney Reduced baseline eGFR Baseline proteinuria	Renal bleeding Urinary leakage Acute kidney injury Renal artery aneurysm Low risk of postoperative cardiovascular complications
	Total nephrectomy	Old age Use of antithrombotics Location in the renal hilum Contralateral kidney normal Multiple small tumors	Loss of contralateral renal function Risk of developing chronic kidney disease Decreased overall survival

## Data Availability

Dataset available on request from the authors.
